# Rebuilding the Gut Microbiota Ecosystem

**DOI:** 10.3390/ijerph15081679

**Published:** 2018-08-07

**Authors:** Antonella Gagliardi, Valentina Totino, Fatima Cacciotti, Valerio Iebba, Bruna Neroni, Giulia Bonfiglio, Maria Trancassini, Claudio Passariello, Fabrizio Pantanella, Serena Schippa

**Affiliations:** Department of Public Health and Infectious Diseases, Section of Microbiology, Sapienza University of Rome, Rome 00185, Italy; antonella.gagliardi@uniroma1.it (A.G.); valentina.totino@uniroma1.it (V.T.); cacciotti.fatima@gmail.com (F.C.); valerio.iebba@uniroma1.it (V.I.); bruna.neroni@uniroma1.it (B.N.); giulia.bonfiglio@gmail.com (G.B.); maria.trancassini@uniroma1.it (M.T.); claudio.passariello@uniroma1.it (C.P.); fabrizio.pantanella@uniroma1.it (F.P.)

**Keywords:** gut microbiota, eubiosis, dysbiosis, therapeutic strategy

## Abstract

A microbial ecosystem in which bacteria no longer live in a mutualistic association is called dysbiotic. Gut microbiota dysbiosis is a condition related with the pathogenesis of intestinal illnesses (irritable bowel syndrome, celiac disease, and inflammatory bowel disease) and extra-intestinal illnesses (obesity, metabolic disorder, cardiovascular syndrome, allergy, and asthma). Dysbiosis status has been related to various important pathologies, and many therapeutic strategies aimed at restoring the balance of the intestinal ecosystem have been implemented. These strategies include the administration of probiotics, prebiotics, and synbiotics; phage therapy; fecal transplantation; bacterial consortium transplantation; and a still poorly investigated approach based on predatory bacteria. This review discusses the various aspects of these strategies to counteract intestinal dysbiosis.

## 1. Introduction

Bacteria (bacteriome), fungi (mycome) [1], and viruses (virome) [2] live together in a harmonic and dynamic equilibrium in the intestinal tract. Although long ignored, viruses play a relevant role in the intestinal ecosystem: 90% of the intestinal virome consists of bacteriophages [3], while the remaining 10% encompasses several plant and animal viruses that are constantly introduced with food. This microbial community begins to colonize the body before delivery and lives in the body in a mutualistic relationship until death. The intestinal microbial community contributes to nutrient metabolism, calibrates metabolic functions, educates/stimulates the immune system, maintains community integrity, and defends the host from pathogens [4,5]. The set of bacterial genomes coexisting with the host is called microbiome. Its coding capacity is 150-fold higher than that of the human genome [6], providing functional features that humans have not evolved. The sum of the human genome plus the contribution of the microbiome is called hologenome, which determines the metabolic characteristics of the organism [6]. Nobel laureate Eli Metchnikoff (1845–1916) said that “the majority of diseases begin in the digestive tract when “good” bacteria are no more able to control “bad” bacteria”, calling this condition dysbiosis. Gut dysbiosis has been linked to several pathologies (inflammatory bowel disease, celiac disease, obesity, metabolic disorder, etc.) [7], and the list continues to grow. The scientific community has now recognized the importance of maintaining a balanced gut microbiota to maintain a healthy status. To this purpose, several strategic therapies to restore and/or to maintain the eubiotic state of the microbic intestinal ecosystem are being studied. After overviewing gut composition and factors impacting equilibrium, this review will focus on therapeutic strategies to restore the gut microbiota ecosystem. Particularly, the administration of probiotics, prebiotics and synbiotics, phage therapy, fecal transplantation, bacterial consortium transplantation (BCT), and approaches based on predatory bacteria will be discussed. 

## 2. Gut Microbiota Composition

The variety and availability of adhesion sites enables the host genome to control the first colonizing bacteria, which modulates the gene expression of host adhesion sites, thereby shaping an intestinal habitat that will support the colonization of related/not competitor species [7,8]. Until recently, it was believed that fetus development occurred within a sterile uterus [9]. This was the dogma, and any microorganism in the uterine cavity was seen as dangerous for the fetus. However, increasing evidence indicates that the fetus develops in an environment that is not entirely germ-free. Many microbial species have been detected in the umbilical cord [10], the amniotic fluid [11,12,13,14,15], and the fetal membranes [12,13,14,15,16] in apparently normal pregnancies without any indication of inflammation or pathology. After birth, the infant acquires microbes from the environment, food, and nearby people. In the first month of life, gut microbiota is less stable, and its biodiversity will increase over time [17]. Parallel to microbial colonization, the human immune system must learn to tolerate the large quantity of antigens present in the environment. Colonization in the early life stages occurs in conjunction with the development, expansion, and education of the immune system. This indicates that during the first colonization steps, factors with a negative impact on microbiota composition could be prognostic of several diseases that may develop in later years. The delivery mode (vaginal delivered babies or Cesarean section delivery) [18,19,20], as well as nutrition (breast or artificial milk) [21,22] are factors that strongly impact the gut microbiota composition. Gut microbiota evolves rapidly and stabilizes at approximately 3 years old [23,24,25]. Factors impacting human gut microbiota development strongly influence baby growth and adult life [26]. At the taxonomic phyla level, a healthy microbiota in adult humans is principally composed of *Firmicutes* and *Bacteroidetes*, which together represent approximately 70% of the total microbiota; *Proteobacteria*, *Verrucomicrobia*, *Actinobacteria*, *Fusobacteria*, and *Cyanobacteria* can also be found, although at lower percentages [27,28]. Obligate anaerobes dominate and exceed by two logs the facultative anaerobes and by three logs the aerobes. At the taxonomic level of species, the gut microbiota composition changes from individual to individual [8,9] and is comparable to a fingerprint. The distribution and abundance of microbiota species/groups diverges considerably in different intestine districts and depends on gastric acid secretion, gastrointestinal peristalsis, mucosal secretion of IgA, as well as on the individual’s immune characteristics and environmental influences [27,28,29]. An increase in microbial density and species biodiversity is observable along the gastrointestinal tract proceeding in the caudal-cervical direction. Differences in gut composition are also observable between the intestinal lumen and the mucosal surface [30]. *Bacteroides*, *Bifidobacterium*, *Streptococcus*, *Enterobacteriaceae*, *Enterococcus*, *Clostridium*, *Lactobacillus*, and *Ruminococcus* are the predominant genera in the intestinal-lumen, while *Clostridium*, *Lactobacillus*, *Enterococcus*, and *Akkermansia* are predominant in the mucosa-associated surface [31]. The mucosa-associated microbiota plays a very important role in maintaining homeostasis, given its proximity to the intestinal epithelium and the underlying mucosal immune system [7]. This microbiota may play an important role in maintaining host cellular homeostasis or in triggering inflammatory mechanisms.

Once established, the composition of the gut microbiota remains stable throughout adult life. Some differences between the gut microbiota of elderly and young adults [32] are observable, primarily concerning the predominance of the *Bacteroides* and *Clostridium* genera in elderly and *Firmicutes* in young adults [33]. Three variants of the human intestinal microbiota have been proposed and classified as enterotypes according to/based on the variation in the levels of one of the three genera: *Bacteroides* (enterotype 1), *Prevotella* (enterotype 2), and *Ruminococcus* (enterotype 3). These three variants appear to be independent from body mass index, age, sex, or nationality [34,35]. 

The distal tract of the human gut is considered an anaerobic bioreactor with a metabolic activity comparable to that of the liver, and for that reason the microbiota could be considered a real organ with specific functions [36]. An organ that consumes, conserves, and redistributes energy goes under physiologically important chemical transformations and is able to maintain and repair itself through self-replication [7]. Like all human organs, the “microbiota organ” has important functions by regulating correlated physiological systems, and the host health status is linked to its correct functioning. Microbiota strongly influences various physiological processes: endocrine and metabolic pathways, expansion and regulation of the immune system, the brain in its cognitive functions, and genome epigenetic changes. A well-functioning microbiota organ is directly related to microbiota balance [37]; consequently, the structure and metabolic status of gut microbiota are associated with a healthy status. When a gut dysbiotic status occurs, the microbiota organ does not function properly, and appropriate therapies should be readily prescribed to restore eubiosis. 

## 3. Factors Influencing Microbiota Composition

Many factors influence the gut microbiota composition. Firstly, the mother’s vaginal and intestinal microbiota can affect the fetus microbiota, and the composition and development of the baby’s intestinal microbiota will be strongly influenced by the mode of delivery (vaginal vs. Cesarean) and by feeding (breast milk vs. formula) [38,39]. Furthermore, therapeutic treatments, hygiene levels, exposure to the natural environment, and genetic background, as evidenced by studies on monozygotic twins [40,41], also influence microbiota composition. In adult life, several factors can still disturb gut microbiota balance: food and minor food constituents (contaminants and food additives); prebiotics, probiotics, and synbiotics use; antibiotics and drug intake; and alcohol abuse. Among nondietary factors, age, sex, stress, [42], gastrointestinal disorders, lifestyle, and infective events can also play an important role in the microbiota composition [7]. Unhealthy dietary habits negatively impact gut microbiota composition and could act as a factor triggering diseases with effects on metabolic pathways. High-fat diets, polyunsaturated fatty acids, and meat were associated with an increased risk of Crohn’s disease (CD) and ulcerative colitis (UC) [43]. Inflammatory bowel disease (IBD) risk could be decreased by modulating gut microbiota community structure and/or its metabolome with a vegetarian diet [44,45]. In addition to inflammation shifts, gut microbiota structure is also associated with colorectal cancer, and this appears to be related to diets with copious red meat, promoting an overgrowth of sulfate-reducing bacteria (common colonic inhabitants). Sulfate-reducing bacteria are able to produce genotoxic substances as acid sulfide [46,47,48]. Conversely, a fiber-rich diet increases the production of short-chain fatty acids (SCFAs), such as butyrate, which is beneficial for human colonocytes and has antitumor properties [47]. Mice fed a high fiber diet are protected from pulmonary allergic inflammation through a mechanism that involves the production of propionate during fiber metabolism by gut microbiota [49,50]. The western lifestyle includes a diet high in animal proteins, total and saturated fats, and simple sugars but low in fruits, vegetables, and other fibers. Several studies indicate that subjects assuming western style diets host a major proportion of *Bacteroides* spp. in their gut microbiota, while diets rich in plant polysaccharides are associated with increased amounts of *Prevotella* spp. [51]. Elevated dietary intake of fat meals impacts bile acid homeostasis and colon tumorigenesis [51]. The gut microbiota is able to metabolize these compounds and converts primary bile acids (cholic acid and chenodeoxycholic acid) into secondary bile acids (deoxycholic acid and lithocholic acid) by a C-7 dehydroxylation. This metabolic transformation influences the enterohepatic circulation of bile acids and the absorption of fat at the small intestine level. In the presence of intestinal dysbiosis, this process is less efficient and the ratio of secondary vs. primary biliary acid is significantly reduced as a direct consequence of a significant quantitative reduction of those bacterial species that are able to convert primary bile acids into secondary bile acids [52,53]. Obese human subjects on a high-protein/low-carbohydrate diet were shown to have reduced amounts of intestinal SCFAs and bifidobacteria [54].

## 4. Intestinal Dysbiosis 

When the central mutualistic relationship among microbiota members, metabolic products, and the host immune system is lost in a microbial ecosystem, a condition called dysbiosis occurs. Generally, in a dysbiotic ecosystem, potentially pathogenic microbes take over at the expense of potentially benefic microbes. When dysbiosis takes place, a loss of overall microbial diversity can be observed [42,55,56], and a parallel overgrowth of species named pathobionts, which are genetic variants of the “pathogenic” microbiota, occurs [55,57,58]. Although 40% of microbial genes may be shared in half of the population, indicating a functional microbial nucleus [59], substantial intra-individual and inter-individual variances in gut microbiota composition are present, which complicates the definition of a healthy microbiota. This is also the reason why dysbiosis is not a single condition and can be classified into different forms. Deficiency dysbiosis is a condition characterized by an overall reduction of beneficial bacterial species (such as lactobacilli and/or bifidobacteria), which can occur as a consequence of nonhealthy diets [60] or antibiotic therapies [61], and can be associated with food intolerances consequent to a deficiency in digestive enzymes (intolerance to milk or meat) [62]. Putrefactive dysbiosis, characterized by an increase in putrefactive bacteria (mainly *Bacteroides*), generally results from a diet rich in fat and meat and poor in fibers [63], the metabolization of which can lead to products such as ammonia, amines, and phenols, which could be the cause of symptoms not limited to the gastrointestinal tract but that can also affect the entire body. Dysbiosis is characterized by bacterial overgrowth in the small intestine [64] due to reduced gastric acid production with an excess of bacterial fermentative activity. These subjects are frequently affected by intolerance to gluten or carbohydrates, and their healthy status worsens following carbohydrate consumption. Fermentative dysbiosis often affects irritable bowel syndrome (IBS) patients [65], patients who receive antibiotic treatment, and those who reduce carbohydrate consumption (low fermentable oligosaccharides, disaccharides, monosaccharaides, and polyols (FODMAPs) diet). Susceptibility dysbiosis is associated with a lost tolerance of intestinal microbiota in which genetic causes (leading to abnormal immune responses towards components of the gut microbiota) play an important role and are linked to IBD and other similar diseases [66]. In susceptibility dysbiosis, alterations to the gut microbiota ecosystem are characterized by, a reduced amount of probiotic bacteria, an increase in potentially pathogens microbes (pathobionts) [44], altered motility of the intestine, and bowel inflammation. Fungal dysbiosis, characterized by the overgrowth of *Candida* or other fungal species in the gut microbiota, is promoted by a diet rich in sugar and low in fibers [67]. Additionally, we should take into account the concept of “beneficial and harmful microbes” because we cannot generally speak about beneficial or harmful species; some species could be beneficial or harmful for one person but not for others. We should also always take into consideration the context/habitat because microbes could have different behaviors in different contexts. Our “microbiota organ”, the composition and functioning of which is influenced by the host genotype, environment, and diet, strongly influences the development and functioning of the intestinal tract, as well as distant organs, including the liver, pancreas, and brain [68]. Taking into account that there are limitations in the modification of long-term defined gut microbiota [35], scientific evidence clearly indicates that, when imbalance of gut microbiota occurs, eubiosis, a mutualistic relationship between microbiota members, metabolic products, and the host immune system, should be promptly restored not only to reduce/eliminate local symptoms but also to guarantee a state of general health.

## 5. Rebalance of the Intestinal Ecosystem

Many therapeutic strategies have been developed to re-establish intestinal eubiosis, and new strategies are constantly proposed and investigated. The main and at present best known and most adopted therapeutic strategies include (i) the administration of probiotic bacteria likely to displace potentially pathogenic bacteria and promote a rebalance of the microbial community; (ii) the administration of prebiotics (i.e., formulations of nutrients being preferentially or exclusively metabolized by probiotic bacteria) to favor the overgrowth of probiotic bacteria; and (iii) the administration of probiotics and prebiotics combinations (called synbiotics). More recent therapeutic approaches have been proposed, including phage therapy, fecal transplantation, BCT, and a still poorly investigated approach based on predatory bacteria. All of these strategies share the same goal of replacing harmful microbes with more favorable ones to restore *eubiosis*.

## 6. Probiotics, Prebiotics, and Synbiotics

### 6.1. Probiotics

The International Scientific Association for Probiotics and Prebiotics redefined probiotics as “live microorganisms that, when administered in adequate amounts, confer a health benefit on the host” to exert a wide range of effects [69]. Probiotics can be used both to prevent the onset of dysbiosis when the patient is exposed to predisposing conditions (prolonged antibiotic therapies, intense physical or mental stress, chronic debilitating diseases, etc.) and as therapeutic agents to rebalance an ongoing condition of dysbiosis. Probiotic strains should: belong to species that form normal components of our gut microbiota; belong to the group of microorganisms designated GRAS (generally regarded as safe), even for immune-compromised patients; prove to stay active and vital (for a reasonable period) in the intestinal environment; and resist when exposed to the gastric environment (bile and pancreatic secretions). Human indigenous strains certainly possess adaptive traits, which allow them a stable colonization and more effective and lasting beneficial effects [70]. Beneficial effects of probiotic strains can be categorized as immunological and nonimmunological [71]. Immunological benefits include the activation of local macrophages, an increase in the production of immunoglobulin, the modulation of cytokine profiles, and the induction of hypo-response to food antigens. Nonimmunological benefits include the digestion process, competition with potential pathogens for nutrients and intestinal adhesion sites, pH alterations, and bacteriocins production [71]. Anticancer properties have also been associated with probiotics, which act as anti-mutagens and exert effects at different stages of carcinogenesis [72]. Currently used probiotics include lactic acid bacteria [73], bifidobacteria, enterococci, the yeast *Saccharomyces boulardii*, dairy propionibacteria, *Bacillus* spp., and the Gram-negative *Escherichia coli* strain Nissle 1917 [74].

Lactobacilli are known to be modulators of intestinal inflammation and immune responses. Their administration is recommended in gastroenteric diseases characterized by high levels of inflammation, in diarrhea prevention, in infections treatment caused by enteric pathogens, and in pediatric patients to prevent/treat infant colics. Several studies indicate that the Lactobacillus/human host relationship should be reconsidered [74]. Notably, only a minority of known Lactobacillus species are found to be residents of the human intestinal tract [75], and a large majority of them are allochthone members derived from fermented food [70,74,75]. Bifidobacteria represent 8–10% of the intestinal microbiota and are able to produce vitamins, enzymes, acetic and lactic acids; they also lower the pH of the colon, inhibit pathogens, and have immune activation properties [76]. The oral administration of *Bifidobacterium bifidum* G9-1 appears to suppress the production of specific immunoglobulin E and to promote the IgA response, which is useful for prophylactic treatment in allergic IgE responses [77]. Bifidobacteria are predominant in the microbiota of breastfed babies [55], and their presence is positively correlated with health status. In contrast, the gut microbiota of formula or mixed fed infants is characterized by a significantly reduced prevalence of bifidobacteria and by an increase in Bacteroides species and *Escherichia coli* [55]. Such differences correlate with an increased incidence of colic pain and other disturbances of intestinal origin. *Bacillus subtilis* is able to secrete many extracellular enzymes (a-amylase, arabinase, cellulase b-glucanacase, and DNase), and it is one of the most effective anti-diarrhea therapies [78]. *Escherichia coli* strain Nissle 1917 increases intestinal homeostasis and improves the intestinal barrier, reducing intestinal epithelial cell invasion by several pathogens [79,80,81,82,83]. Finally, bacteria strains belonging to the *Streptococcaceae* family, particularly the two genera *Streptococcus* and *Lactococcus*, as well as the strain *Enterococcus faecium* (ex *Streptococcus*, now separated to from the genus *Enterococcus*), have also been used as probiotics in food and feed. *Lactococcus lactis* is highly resistant against artificial gastric acid and bile juices [84,85]. The probiotic *Lactococcus lactis* is an indigenous species that produces bacteriocins (active on several pathogens) and lactic acid. It also cooperates with the hydrolysis of milk proteins, thus facilitating milk digestion [84,85,86,87]. *Streptococcus thermophilus* has anti-inflammatory properties and helps fight potentially pathogenic bacteria [88]. Gastrointestinal disorders treated with probiotic *Enterococcus* spp. have been evaluated in several hosts (mice, piglets, and humans) [89]. *E. faecium* was shown to affect gut microbiota structure, to regulate immune function, to show inhibitory effects versus enteric pathogens, [90,91] and is a lactic and butyric acid producer. *Enterococcus faecium* is not GRAS, and its use as a probiotic is still questioned. Several strains of the *Enterococcus* genera are associated with infective diseases [92,93,94,95] and could represent a risk for antimicrobial resistance and virulence gene transfers to human strains. Therefore, further benefit/risk evaluations of *E. faecium* use as probiotic should be carried out. *Saccharomyces boulardii* is a yeast probiotic that is resistant to gastric acidity, to proteolysis, and of course to antibiotics [96]. Although the oral administration of *Saccharomyces boulardii* is not able to stably colonize the intestine (it is eliminated within a few days), it is nonetheless able to reach and maintain high concentrations in a short time. These characteristics make it suitable for use during antibiotic treatments. Available data indicate that it promotes eubiosis by facilitating the production of lactic acid and group B vitamins and by preventing the proliferation of harmful yeasts. A recent study showed that the administration of *Saccharomyces boulardii* for four weeks resulted in a significant reduction in the daily number of evacuations and diarrhea in patients with IBS [97]. In recent years, thanks to their safety and effectiveness, probiotics were included not only in dairy products but also in nondairy foods such as fruit juices and cereals [87]. Several recent studies have focused on the utilization of probiotics to prevent antibiotic-associated diarrhea, to limit the use of antibiotics, and to consequently reduce the spread of antibiotic-resistant strains [98,99]. The key messages from these studies are (i) a confirmation that the administration of probiotics helps minimize the prevalence and severity of infectious diseases (as a consequence of the implementation of antimicrobial immune responses and of the general health of the individual); (ii) their specific ability to rebalance the gut microbiota permits their use as the sole treatment in many cases of intestinal disorders, thus significantly reducing the prescription of antibiotics; (iii) even when antibiotics are necessary, the co-administration of probiotics reduces the duration of treatment; (iv) the reduced prescription of antibiotics is certainly potentially associated with a reduced spread of antibiotic resistance, although this is not easy to demonstrate. However, the probiotics in use show limitations, indicating a need to improve the selection and formulation of bacterial strains [100]. Promising results have been obtained in the prevention/treatment of metabolic or inflammatory diseases in preclinical studies conducted on bacterial strains that are different from the classic *Lactobacillus* and *Bifidobacterium* strains [100,101,102]. The next generation probiotics includes *Akkermansia muciniphila*, members of *Clostridium* clusters IV, XIVa, and XVIII, and *F. prausnitzii* [103]. Next-generation probiotics must include strains belonging to major gut microbiota groups, and they should be safe and possess potential beneficial effects [104]. *Akkermansia muciniphila* is a strict anaerobe of the phylum *Verrucomicrobia*, which is a mucin-degrading microbe that is related to a healthier metabolic status. *Akkermansia muciniphila* has been demonstrated to be significantly decreased in obesity, in subjects with fat metabolism disorders, diabetic subjects, and subjects with other metabolic disorders [103,105]. Studies [103,105] on the outcomes of a high fat diet on metabolic factors and gut microbiota structure over time highlight a decrease *of A. muciniphila*. Li and collaborators found that *A. muciniphila* had the ability to reverse atherosclerotic injuries, improve gut barrier restoration, and reduce metabolic endotoxemia-induced inflammation [106]. The oral administration of another new probiotic candidate, *Bacteroides uniformis* CECT 7771, to high fat diet-fed mice indicates an improvement in lipid profiles, leptin and glucose level, and an increase in TNF-α production after LPS stimulation [107]. *B. uniformis* CECT 7771 did not present unfavorable effects on health status; however, an additional search needs to be performed in humans [108]. *Clostridium* spp. belonging to clusters IV and XIVa (also known as the *Clostridium leptum* and *coccoides* groups, respectively) are Tregs inducers in the colon and could be studied as IBD and allergy therapeutic choices [109]. Atarashi and collaborators isolated 17 Clostridia strains belonging to clusters XIVa, IV, and XVIII from a human fecal sample, which were active in Treg cell differentiation. Treg cell differentiation could be the consequence of influenced Foxp3 expression (a gene controlling Treg cell development) by SCFAs produced by this Clostridia strain consortium [110]. Furthermore, fecal samples from IBD patients show a decrease in Clostridia clusters XIVa and IV, indicating a therapeutic potential in the 17-strain cocktail to resolve dysbiosis [110]. *Faecalibacterium prausnitzii*, a species with proven anti-inflammatory properties [111,112], is able to produce butyrate and many SCFAs, which were found to be reduced in Crohn’s disease, obesity, asthma, and major depressive disorders [111]. Its supernatant inhibits the NF-κB pathway in vitro and in vivo [113]. Different animal models, such as a dinitrobenzene sulfate-induced colitis model, dextran sodium sulfate-induced colitis [113], and 2,4,6-trinitrobenzenesulfonic acid induced acute colitis in mice [114], and were used to demonstrate the properties and protective effects of *F. prausnitzii*. Studies have recently focused on engineering probiotic bacteria to create new generation probiotics. These recombinant bacteria are designed to perform specific functions in the gastrointestinal tract: secrete therapeutic molecules (mostly peptides and small proteins) and detect specific signals (including small molecules derived from other bacteria, food, or cancerous or inflamed tissues) [115,116]. More effort should be expended to explain the mechanisms underlying the beneficial effects of probiotic bacteria that have been disclosed during clinical studies. This would provide a stronger basis for the utilization of probiotic bacteria in different applicative fields and could also enhance clinical results by allowing a more rational application of single species endowed with activities related to clinical needs. Moreover, because the cooperating nature of microbiomes appears to be an essential characteristic of the gut microbiota in healthy and disease statuses, studies should develop therapies based on multi-probiotics that are able to influence this network of cooperating organisms and that can ensure a stronger and long-lasting rebalancing effect. Studies carried out with omics approaches should be able to demonstrate a sub-network bacterial consortium that works jointly but in different ways to influence important human physiological process. Paul W. O’Toole and colleagues developed an artificial bacterial consortium that mimics the structure of healthy intestinal microbiota. Starting from fecal samples of healthy donors, and based on the existing literature, 100 different commensal strains were selected with a range of abundance values in the microbiota. This live bio-therapeutic association of microorganisms has been successfully used for the modulation of the intestinal microbiota of elderly people [116]. A combination of 17 *Clostridium* strains of human origin were shown to reduce the severity of induced allergic colitis in rodents; these effects were mediated by the activation of Treg cells; however, the identity of bacterial products implicated in this activation remains unclear [110]. The mixture VSL#3 (composed of four lactobacilli strains: *Lactobacillus casei*, *Lactobacillus plantarum*, *Lactobacillus bulgaricus*, and *Lactobacillus acidophilus*; three from bifidobacteria: *Bifidobacterium longum*, *Bifidobacterium breve*, and *Bifidobacterium infantis*; and *S. thermophilus*) has showed positive effects in UC treatment [117,118,119], whereas indications for probiotic efficacy in CD are low [120]. Ecologic^®^Tolerance/Syngut^TM^ is another mixture containing four probiotic strains (*Bifidobacterium lactis* W51, *L. acidophilus* W22, *L. plantarum* W21, and *Lactococcus lactis* W19). This consortium appears to reinforce gut barrier function, show a beneficial impact on post-immunological provoked stress, and inhibit and stimulate Th2 and IL-10 levels, respectively, therefore offering useful effects in food intolerance patients [121]. Additionally, multispecies probiotics relieved IBS symptoms and modulated microbiota composition [122]. The dose needed to ensure the clinical efficacy of probiotics is variable; in general, products containing probiotics must have a minimum number of cells viable between 10^6^ and 10^8^ colony-forming units per gram (CFU/g) of the product final or 10^8^–10^10^ CFU/day (considering 100 g or 100 mL of ingested food) [123]. Although the long use of probiotics, as well as data from in vitro and in vivo studies, corroborates the notion that probiotics are safe, some case reports note evidence of several risks, including systemic infections, altered metabolic pathways, intensified immune stimulation, gene transfer, and gastrointestinal disorders. More studies are required to accurately define the occurrence and severity of unfavorable events linked to probiotics [124]. 

### 6.2. Prebiotics and Symbiotc Formulations

The concept underlying the use of prebiotics was first introduced in 1995 [125]. According to the Global Guidelines of the World Gastroenterology Organization, prebiotics are nondigestible substances taken by the human host that, when taken in adequate amounts, produce beneficial physiological effects on the host by stimulating, in a selective manner, the growth and metabolic activity of a limited number of beneficial indigenous bacteria (bifidobacteria and lactic acid bacteria) [107,108,109]. Prebiotics are considered a specific fuel that indigenous probiotic bacteria can utilize to grow. Prebiotics are primarily dietary components of foods (mostly nonstarch polysaccharides and oligosaccharides) used as enrichment ingredients. Most commonly known and characterized prebiotics include fructo-oligosaccharide supplements (FOS), galacto-oligosaccharides, inulin (also able to increase calcium absorption), lactulose (a synthetic disaccharide used as a drug for the treatment of constipation and hepatic encephalopathy), and breast milk oligosaccharides [126,127]. However, inulin supplementation modulates metabolic endotoxemia and inflammation in women with type 2 diabetes [128,129]. These substances are frequently included in synbiotics formulations containing probiotic bacteria to promote their rapid growth in the intestinal environment. FOS are able to cross the digestive lumen, undigested and unabsorbed, to reach the ascending colon unmodified, where they will be selectively metabolized by the resident probiotic component of the microbiota. Their digestion causes a significant decrease in pH, creating an unfavorable habitat for putrefactive bacteria (clostridia) growth. Consumers do not have sufficient technical knowledge to choose the correct FOS without medical supervision. Finally, of note are postbiotics, which are bioactive microbial metabolites derived from heat-killed microbes that present positive effects on human functioning by interacting with the immune system and presenting anti-inflammatory outcomes [130]. Structural bacterial constituents may be promising candidates as inducers of beneficial effects in humans. These are also attractive because they can be part of treatments encompassing non-viable bacterial cells [130]. Because the microbiota structure/composition is comparable to a fingerprint and there are different levels and types of dysbiosis, the correct use of probiotics/prebiotics/synbiotics should consider prior knowledge of the type of dysbiosis to proceed with a targeted treatment for the patient. The positive effect of probiotic, prebiotic, or synbiotic treatment could depend on the individual’s pathology, as indicated by systematic reviews considering different milieus [131,132]. Results of studies conducted thus far on pro/prebiotics are very variable and reflect the diversity of the tested probiotic strains, as well as the diversity of the populations examined. Prebiotics appear to be promising therapeutic options for gastrointestinal diseases; however, further studies are needed with larger study populations to establish their effectiveness, modalities, and treatment durations. Large-scale studies, specifically well-designed randomized controlled trials, are essential to demonstrate the security and effectiveness of these supplements. Furthermore, few studies have been carried out on gastrointestinal discomfort following treatment with probiotic, prebiotic, and synbiotics, such as high osmotic pressure, flatulence, and bloating [133]. 

## 7. Diet Approach

### Diet Approach to Modulate Gut Microbiota

The Mediterranean and Atlantic diets should be distinguished from the Western diet. The Mediterranean and Atlantic diets are both considered to preserve a good health status [134]. The Mediterranean diet is an assortment of habitual eating behaviors followed by people in the countries contiguous to the Mediterranean Sea. Significant protection from chronic degenerative diseases has been provided by observing the Mediterranean diet [134,135,136,137]. The Atlantic diet has been associated with metabolic health and lower mortality from coronary diseases and some cancers [116]. The three components of the Atlantic diet include Vitamin B, omega 3 fatty acids, and iodine, which may bring health benefits to consumers in the Atlantic area [138]. Diet has an immediate impact on microbiota composition. This can be synthetically described in terms of an increase or decrease in representative groups of species, as well as of a significant modification in the metabolites released in the environment (and in part absorbed by the host). Nondigestible carbohydrates are fermented by the intestinal saccharolytic microbiota, thereby producing SCFAs. The types and amount of carbohydrates that we consume quantitatively and qualitatively influence the single bacterial species. To increase bacterial fermentation and SCFAs production, diet adjustment is a very attractive and safe therapeutic strategy. Furthermore, when adopting a dietary approach, the contribution of micronutrients should be considered as an important factor influencing gut microbiota composition. Micronutrients (zinc, vitamins D and A, folate) deficiency in early life may influence the maturation of the gut microbiota and its interaction with the host, with effects in adolescence and adult life [139,140]. Additional information about gut micronutrient synthesis and its impact on microbiota composition and functions is necessary to improve the current understanding of the role of micronutrients. Studies investigating the impact of the microbiota on obesity and other pathologies should take into account the impact of micronutrient deficits [141]. A study using chickens as an animal model demonstrated that zinc deficiency provokes changes in the microbial ecosystem composition and in metabolic profiles with a decrease in SCFAs [141]. Epigenetic processes influence microbiota/host communications; folate is an essential donor of the methyl group in methylation reactions that are associated with epigenetic changes [139]. 

A diet-based approach to modulate the microbiota should consider the effect of long-term diets [141,142]. Recent studies have highlighted important differences in the ability to modulate microbiota composition in long-term and short-term diets. In short-term diets, changes are significant and rapid, but the magnitude of changes is modest and insufficient to relocate individuals from one enterotype to another [35]. In contrast, long-term diets are adequate to relocate enterotypes [35]. If an enterotype is shown to be causative/linked to a disease, long-term dietary interventions could represent a good strategy to help [35]. Among diet interventions, a feeding regime with a low content of fermentable oligosaccharides, disaccharides, monosaccharaides, and polyols (FODMAPs) was shown to reduce gastrointestinal symptoms in patients with IBS in less than 48 h [142]. 

In IBS patients who experienced a low FODMAPs diet, carbohydrates fermentation is reduced, and a decrease in luminal osmolarity and gas generation (e.g., hydrogen) is observed. Consequently, typical IBS symptoms of gas and bloating are reduced/eliminated [142]. However, more studies should be carried out on the effects of a long-term low FODMAPs diet. It should be borne in mind that FODMAPs, especially oligosaccharides, play an important role in stimulating the growth of beneficial bacterial groups. The long-term assumption of a low FODMAPs diet could have unpredictable effects on the composition of gut microbiota. 

Diet is an easily modifiable factor and is consequently a very attractive therapeutic approach to modulate gut microbiota. Several functional foods are proposed at present, but a diet fitting all subjects is impossible; personalized functional foods are instead required. Metabolic profiling technologies provide valid support for the improvement of functional foods. The existence of high inter-individual variability indicates that a more personalized approach, accompanied by personalized functional foods, is the way forward. 

Medicines derived from microbiota should be employed to treat gut dysbiosis. The development of functional foods can be supported by metabolic profiling. This can start with food composition and by looking for biomarkers that are helpful to trace ingested food [143]. Randomized, clinically controlled dietetic interventions to shape the gut microbiota of humans have been described. Results indicate that energy-restricted foods rich in fiber and vegetables guarantee microbial changes in the gut and present health advantages [143,144,145]. Specific dietetic treatments, alone or in addition to combinations of probiotic species, could represent a potentially interesting tool to improve public health [43]. Furthermore, we should consider the subjects that do not respond to diet treatments. This could depend on several factors, such as the subject age and microbiota composition before diet treatment [144,145]. Additionally, some bacterial clusters persist unaltered by alimentary modification, likely because they are able to consume a wide range of dietary resources and are able to adapt/change their metabolism as a function of the environmental/nutritional change [146]. Additionally, in a diet approach to rebuild the gut microbiota, we must have prior knowledge of the type of dysbiosis to establish personalized/targeted treatments.

## 8. Fecal Microbiota Transplantation 

The use of feces for therapeutic purposes is not a recent discovery. As early as 4th century China, suspensions of feces were used to treat food poisoning. Following suggestions from the Bedouins, during the Second World War in Africa, German soldiers adopted the consumption of fresh camel feces as a remedy for bacterial dysentery [147]. In 1958, Ben Eiseman, an American physician, treated four patients affected by pseudomembranous colitis with fecal microbiota transplant (FMT) [148]. The first successful treatment of a *Clostridium difficile* infection (CDI) by FMT was documented in 1983 [149]. While all previously discussed approaches are substantially comparable to conventional therapies, FMT resembles an organ transplant: the transplant of the microbiota organ. Currently, FMT is a treatment for *Clostridium difficile* diarrhea that is unresponsive to standard antibiotic therapy [150]. Microbiological investigations revealed that, following FMT, a rapid change of fecal microbiota composition is observed in the receiving patient and that the microbiota becomes similar to that of the healthy donor. Such changes are maintained for at least up to 24 weeks [151]. Although very simple in its general traits, FMT requires care, as with any other organ transplant, especially with respect to donor selection. All analysis addressed to evaluate the risk of transmission of any infectious disease must be carried out on the selected donor. According to protocols presently in use, any potential donor is subjected to blood and stool sampling 4–5 days before the collection of the feces to be processed for transplantation to assess negativity for hepatitis viruses (A, B, and C), HIV, *Treponema pallidum*, *C. difficile*, and common gastrointestinal pathogenic bacteria and parasites. 

In addition, we believe that the eubiotic status of the donor fecal microbial ecosystem should also be evaluated. This could be performed either by species-specific qPCR for species/groups of bacteria, which are indicated by literature to be important for the eubiosis status, or more deeply by next generation sequencing methods. The evaluation of the donor fecal ecosystem eubiosis status should become a must. Several recent studies indicate that changes in microbiota balance could play an active role in the pathogenesis of several diseases rather than being a simple consequence of it [152,153,154]. Experimental data in murine models showed that in genetically predisposed mice, intestinal inflammation can be induced by transferring the intestinal microbiota from mice affected by ulcerative colitis [154,155]. 

An inappropriate donor selection could expose the recipient to several risks, including the modification of the nutritional status and body weight; an alteration in nutrient absorption; or the acquisition of chronic diseases, obesity, diabetes, cardiovascular diseases, or IBD [152,153,154,155], all of which are related to a dysbiosis status of gut microbiota. To overcome many of the logistical difficulties of FMT delivery methods (clyster or nasogastric tube), capsules containing fresh bacterial preparations could prove to be an important step ahead [156]. The extremely high success rates achieved with FMT in the treatment of *Clostridium difficile* diarrhea have catalyzed the attention of a large variety of clinicians and researchers for its potential applications in the treatment of many different pathologies characterized by the presence of intestinal dysbiosis [157,158,159,160,161], such as Crohn’s disease [159], ulcerative colitis [158,160], obesity [161], and several metabolic disorders [162,163,164,165,166]. 

The substitution of the microbiota organ by FMT could potentially impact obesity [161]; however, concerns regarding its utilization derive from the potential risk of transmitting secondary diseases. Results presently available on the therapeutic use of FMT are nevertheless contradictory: patients with ulcerative colitis appear to respond better than those with Crohn’s disease [158,160]. A consensus conference of experts from different countries recently outlined methods and indications for the FMT procedure in managing CDI, suggesting the implementation of FMT centers for CDI treatment; it also encouraged the translation of scientific and technical information to study the potential of FMT in different pathological fields, including CD, UC, and IBS [162]. Given the astonishing success obtained with fecal transplantation in the treatment of *C. difficile* diarrhea, we could suppose that dysbiosis linked to long-term antibiotic therapies and, generally speaking, dysbiosis showing a strongly unbalanced microbiota ecosystem, which do not respond to treatment with single probiotic strains, require a more comprehensive treatment. This would imply the entire replacement of the “microbiota organ”. However, thorough studies on FMT, such as accurate trials and cohort studies with control groups, are desirable to corroborate its long-term effectiveness and safety [163]. Microbiota rebalanced by well-defined microbial communities comprising gut bacteria could represent an alternative to FMT [164] (see paragraph: Bacterial consortium transplantation).

## 9. Bacterial Consortium Transplantation 

A specific modulation of the intestinal ecosystem could be performed with BCT. A recent study showed that in mice with antibiotics-induced intestinal dysbiosis, a complete recovery of the microbial community was obtained either with FMT or BCT, indicating that the effects of BCT are comparable to those of FMT [165,166]. The use of characterized microbial populations of specific fecal bacteria may be developed to substitute FMT [164]. An artificial bacterial combination of 33 different purified gut bacteria isolated from a healthy donor (stool substitute (RePOOPulate): *Acidaminococcus intestinalis*, *Bacteroides ovatus*, *Bifidobacterium adolescentis* (two strains), *Bifidobacterium longum* (two strains), *Blautia* product, *Clostridium cocleatum*, *Collinsella aerofaciens*, *Dorea longicatena* (two strains), *Escherichia coli*, *Eubacterium desmolans*, *Eubacterium eligens*, *Eubacterium limosum*, *Eubacterium rectale* (four strains), *Eubacterium ventriosum*, *Faecalibacterium prausnitzii*, *Lachnospira pectinoschiza*, *Lactobacillus casei/paracasei*, *Lactobacillus casei*, *Parabacteroides distasonis*, *Raoultella* sp., *Roseburia faecalis*, *Roseburia intestinalis*, *Ruminococcus torques* (two strains), *Ruminococcus obeum* (two strains), and *Streptococcus mitis*) was able to treat recurrent CDI [167]. Bacterial consortiums are accurately defined and reproducible and may warrant standardization (the proportions of each bacterium in the BCT) or even personalized preparation based on different levels and/or types of dysbiosis. Furthermore, patient safety could be improved because the bacterial combination can be controlled for pathogen microorganism presence [167]. In this view, BCT could be an effective and safer alternative to FMT to modulate the intestinal microbiota dysbiosis.

## 10. Phage Therapy 

Phages are viruses that infect bacteria, representing approximately 90% of the human virome and having a great influence on bacterial populations of microbial communities. Phages have a great therapeutic potential: they could be used either for antimicrobial purposes (alternative to antibiotics) or to modulate the composition of microbial communities [168]. In addition, genetically modified phages could be used as “gene carriers” for the biosynthesis and degradation of nutrients as well as for genetic modulation of the intestinal microbiota. 

Given the presence in our microbial ecosystem of phages, the risks of this therapeutic approach do not appear to be high. The potential of bacteriophages as anti-infectious agents was recognized several decades ago; protocols for bacteriophage therapy have been developed since the early 20th century and applied mainly in Eastern Europe and Russia [169,170,171,172]. Unfortunately, these studies were not designed according to the criteria accepted by the international community for medical research [152] and, consequently, new studies are needed together with a modification of the current European Regulatory Framework, which is an obstacle to the utilization of phage therapy.

Suspensions of phages can be prepared for both local or systemic therapy [173]. Notably, phages amplify exponentially following administration. Furthermore, the kinetic of amplification is not constant and depends on the concentration of susceptible bacteria and to the immune responses of the human host. These factors work differently in acute and chronic infections, making the exact dosing and timing of administration problematic as well as of primary importance [174,175,176,177]. 

Despite an extremely long experience with phage therapy as a result of its constant use in Eastern Europe since the beginning of the 20th century [178,179], essential data for the approval of phages as antibacterial drugs by the FDA and EMA are still needed, and studies to address these points are necessary.

## 11. Predatory Bacteria 

In their natural ecosystems, bacteria are subject to predation not only by bacteriophages but also by predatory prokaryotes [180]. Predation is just one of the strategies adopted by bacteria to interact with other bacterial species. It was reasonably proposed that the term predator should be applied to those bacteria that actively hunt and kill their prey and consume their macromolecules as nutrients [160]. Predators and prey are present in all ecosystems, and the ratio of their relative abundance is important to maintain bionetwork balance. Predatory bacteria are in general smaller than their prey, which enables the predator to penetrate inside the prey, kill it from the inside, and replicate. It is remarkable that among predatory bacteria, distinct predatory behaviors have evolved; among these, the commonly indicated epibiotic predation is a strategy that does not require intracellular replication [181,182]. 

Although predatory bacteria play a relevant role by controlling and affecting bacterial populations in a wide range of environments, and despite the fact that they are known to be almost ubiquitous, only a minority of these bacteria have been studied in some detail for their potential applications [182,183,184,185,186,187]. 

Our research group recently showed that the intestinal mucosa ecosystem is normally colonized by the bacterial predator *Bdellovibrio bacteriovorus* [188], and when dysbiosis occurs, with an overgrowth of Gram-negative bacteria, the predator strain is not detectable. 

According to our findings, *B. bacteriovorus* is not equally distributed in the various districts of the intestinal tract. We found that it is more prevalent in the duodenum (the site in which the mucosal surface is normally less colonized by bacteria) and becomes progressively less distally prevalent toward the rectum zone. The fact that some predatory bacteria are normally isolated from the microbiota of healthy humans indicates that these bacteria are not pathogenic and suggests that they could contribute to the homeostasis of these environments. By preying on Gram-negative bacteria, attempting to massively colonize the intestinal mucosa, *B. bacteriovorus* exerts its role of controlling and shaping indigenous bacterial populations. 

These observations allow speculation on the possible use of predatory bacteria as a tool to re-equilibrate a *dysbiotic* gut microbiota in which Gram-negative bacteria predominates. The use of *B. bacteriovorus* to restore *eubiosis* in the gut microbiota has already been attempted with success in birds [189]. Considering the potential future application of predatory bacteria, it must not be forgotten that bacterial predation has been going on for millions of years, in which the other players (the prey) have evolved their own defensive strategies. Experimental data demonstrate that the introduction of predators in an ecosystem frequently causes the evolution of several defensive characteristics in the prey. It deserves consideration that many of these mechanisms also function as virulence factors and, consequently, the presence of predators could act as a selective factor inducing an increase in the undesirable pathogenicity and virulence of the microorganisms colonizing the environment [190,191]. 

This latter datum certainly indicates that we still need to check the effectiveness of the predator strategy, as well as its potential applications and risks. Notably, predators exist in natural ecosystems and, as recently evidenced, also in the human gut and possibly in other microenvironments of the human body. In the human gut, their presence was shown to decrease under detection limits in dysbiosis. 

Although these data are clearly suggestive of the possible therapeutic application of predatory bacteria, we believe that their use should be based on adequate and accurate experimental data to precisely determine the doses to be administered. We believe that predatory bacteria could otherwise prove deleterious, just as antibiotics when used inappropriately. The use of predatory bacteria could be considered when over-colonization by Gram-negative bacteria of the intestinal mucosae occurs, as when it happens in IBD or celiac disease.

## 12. Conclusions

The structure of the gut microbiota undergoes significant fluctuations over the course of a lifetime; these modifications are frequently associated or accompanied by undesirable effects on human health. Fluctuations are influenced by several factors such as lifestyle, stress, nutritional factors, and antibiotics. Strategies to counterbalance these harmful fluctuations were shown to be effective in reducing symptoms and sometimes curing some of these pathologies. 

Many studies are currently underway to determine how to prescribe personalized therapies to re-balance the intestinal microbial ecosystem. The great interest in this field demonstrates the importance of the maintenance of the intestinal microbial balance and also shows that there is no universal cure suitable for all individuals. The therapeutic strategies actually in use showed limits, and new probiotic candidates (Table 1) show promising results [100]. Moreover, considering the collaborating nature of microbes living in gut ecosystem, studies should primarily focus on the improvement of treatments based on multi-probiotics (Table 2). Additionally, the prescription of personalized therapies that take into account the various types of dysbiosis and the individuality of gut microbiota structure must cautiously consider the gut microbiota of each patient by evaluating all the information from integrated “omic” platforms. These platforms will enable the characterization of microbial communities by examining not just what they are, but also what they do, together with their genetic potential. Strategies based on prebiotics, probiotics, and lifestyle adjustment are already widely available but need to be implemented by specialized medical supervision and need to be directed to the specific dysbiosis present. Innovative strategies, such as FMT, BCT, predatory bacteria therapy, phage therapy, and next generation probiotics, need to be further studied before being routinely applied; they nevertheless appear fascinating, and potentially present great efficiency. 

## Figures and Tables

**Table 1:** New probiotic candidates.

Strategy	Disease	Outcomes Should Be	Bibliography
Akkermansia muciniphila	Obesity Metabolism disorders Diabetic subjects	Re-equilibrate gut microbiota dysbiosis Reverse atherosclerotic injuries	[103,105,106]
Faecalibacterium prausnitzii	Dysbiotic gut microbiota	Re-equilibrate gut microbiota dysbiosis NF-κB pathway inhibition Protective effects Short chain fatty acids (SCFAs) production	[111,112,113,114]
Predator bacteria (Bdellovibrio bacteriovorus)	Gram negative infections Dysbiotic gut microbiota (with predominance of gram negative bacteria)	Re-equilibrate gut microbiota dysbiosis with gram negative overgrowth	[160,161,162,163,164,165]
*B. uniformis* CECT 7771	High fat diet-fed mice	Improvement in lipid profiles, leptine, and glucose level. Increase in TNF-α production	[107,108]
Recombinant bacteria	Designed to perform specific functions: Produce therapeutic molecules Detect specific signals	Re-equilibrate gut microbiota dysbiosis	[102,103]
Phage therapy	Infections Gut dysbiosis	Re-equilibrate gut microbiota dysbiosis Therapy have been developed mainly in Eastern Europe and Russia Modified Phages: “gene carrier”	[147,148,149,150,151]

**Table 2:** Multi-probiotics.

Strategy	Disease	Adverse Events	Bibliography
Bacterial consortium transplantation (BCT) Stool Substitute (RePOOPulat)	Induced gut dysbiosis in mice CDI	No	[146,166,167]
VSL#3	Dysbiosis induced by antibiotic Ulcerative colitis Pathogen infection Atopic dermatitis	No	[118,192,193]
Ecologic® Tolerance/SyngutTM	Food intolerance	No	[121]
Ecologic AAD	Diarrhea induced by amoxicillin	No	[194]
17 Clostridium strains of human origin	Rodents with induced allergic colitis	No	[104]
Fecal Microbial Transplantation	CDI Induced colitis model	Transmission risk of IBD Infectious disease Autoimmune disease	[148,149,150,151,152,195]

## Abbreviations

The following abbreviations are used in this manuscript:

**FMT**	Fecal Microbial Transplantation
**BCT**	Bacteria Consortium Transplant
**SCFA**	Shorty Chain Fatty Acid
**CDI**	*Clostridium difficile* infection
**NGS**	Next Generation Sequences
**IBD:**	Inflammatory Bowel Disease
**CD**	Crohn’s disease
**UC**	Ulcerative colitis
**FODMAP**	Fermentable Oligosaccharides, Disaccharides, Monosaccharaides, and Polyols
**GRAS**	Generally Regarded As Safe
